# Patients’ knowledge on cardiovascular risk factors and associated lifestyle behaviour in Ethiopia in 2018: A cross-sectional study

**DOI:** 10.1371/journal.pone.0234198

**Published:** 2020-06-04

**Authors:** Lemma B. Negesa, Judy Magarey, Philippa Rasmussen, Jeroen M. L. Hendriks

**Affiliations:** 1 Adelaide Nursing School, The University of Adelaide, Adelaide, Australia; 2 College of Health and Medical Sciences, Haramaya University, Harar, Ethiopia; 3 Royal Adelaide Hospital, College of Nursing and Health Sciences, Flinders University and Centre for Heart Rhythm Disorders, Adelaide, Australia; 4 Department of Medical and Health Sciences, Linköping University, Linköping, Sweden; Universidad del Desarrollo, CHILE

## Abstract

**Background:**

Cardiovascular disease (CVD) is posing a major public health challenge globally. Evidence reports significant gaps in knowledge of cardiovascular risk factors among patients with CVD. Despite the growing burden of cardiovascular disease in developing countries, there is limited data available to improve the awareness of this area, which is crucial for the implementation of prevention programs.

**Methods:**

A cross-sectional survey was conducted in two referral hospitals in Eastern Ethiopia from June-September 2018. Outpatients with a confirmed diagnosis cardiovascular conditions were eligible for participation in the study. A convenience sampling technique was used. The primary outcome of the study was knowledge of cardiovascular risk factors among patients with cardiovascular disease. The knowledge of cardiovascular disease risk factors was measured using a validated instrument (heart disease fact questionnaire). A score less than 70% was defined as suboptimal knowledge. Multivariable linear regression was used to examine the relationship between knowledge of cardiovascular risk factors and explanatory variables.

**Results:**

A total of 287 patients were enrolled in the study. Mean age was 47±11yrs and 56.4% of patients were females. More than half of patients (54%) had good knowledge on cardiovascular risk factors (scored>70%), whilst 46% demonstrated suboptimal knowledge levels in this area. Urban residency was associated with higher cardiovascular risk factors knowledge scores, whereas, never married and no formal education or lower education were identified as predictors of lower knowledge scores. There was no statistically significant association between knowledge of cardiovascular risk factors and actual cumulative risk behaviour.

**Conclusion:**

Almost half of CVD patients in Ethiopia have suboptimal knowledge regarding cardiovascular risk factors. Residence, education level and marital status were associated with knowledge of cardiovascular risk factors. Implementation of innovative interventions and structured, nurse-led lifestyle counselling would be required to effectively guide patients in developing lifestyle modification and achieve sustainable behaviour change.

## Introduction

Cardiovascular disease (CVD) remains a global major cause of death [1] and represents a significant disease burden in populations around the world. The global burden of disease studies reported an estimated 422.7 million cases of CVD, causing 17.92 million deaths worldwide in 2015 [1]. Developing countries are facing a high burden of CVD whilst awareness of disease and associated risk factors is limited [2, 3]. Those living in poverty and especially those in low-income countries are significantly more impacted by CVD [4]. Moreover, findings show that the prevalence of CVD is increasing and posing a public health challenge in developing countries [1, 5]. High blood pressure is of major influence in the increasing CVD burden in these countries [1]. For most patients with hypertension it is uncontrolled which causes further cardiovascular (CV) complications [6]. Hypertension affects more than 1.3 billion people worldwide and one third of adults have the condition [7, 8]. The number of adults with hypertension in 2025 is predicted to increase by about 60% [9]. Moreover, the total number of individuals with hypertension is increasing rapidly to epidemic levels with a projected 125.5 million individuals affected by 2025 in Sub-Saharan Africa [10].

From an epidemiologic view on disease prevalence, Ethiopia is in epidemiologic transition from predominantly infectious diseases to chronic diseases. CVD is a major public health challenge in Ethiopia. The overall prevalence of hypertension among the Ethiopian population is 19.6%, and is higher among the urban population (23.7%) [11]. In 2015, ischemic heart disease was the first leading causes of age standardised death rates and fourth leading causes of age standardized disability adjusted life years with rates of 141.9 and 2535.7 per 100,000 population respectively [12]. The increasing prevalence of CVD in developing countries is related to unhealthy lifestyle behaviours. Except few region based studies, evidence on CV risk behaviours is scarce in Ethiopia. Findings from the Southern part of the country show that 10.8% of CV patients smoke cigarettes, 12.1% drink alcohol and 73.9% don’t do any physical activity [13]. A study performed in the capital of Ethiopia reported 68.6% of hypertensive patients don’t exercise, 14.1% smoke cigarette, 25.2% drink alcohol and 30.9% don’t adhere to healthy diet [14].

According to the health belief model, knowledge regarding health behaviour is a strong modifying factor for healthy lifestyle, however it should be combined with other factors such as good perceptions, positive health attitudes and many other conditions such as socioeconomic factors [15]. Studies also have revealed knowledge of specific risk factors is associated with healthy behaviour, however, knowledge alone does not motivate behavioural change [16–19]. The Heart disease fact questionnaire which was designed and validated by Wagner et al. (2005) and has been commonly used for the assessment of knowledge of CV risk factors knowledge [20].

There is limited research regarding the knowledge of CV risk factors in developing countries [3, 21–23]. The majority of adults in Sub-Saharan Africa fail to name even one CV risk factor, [22] and in Nigeria almost 50% have poor knowledge about CV risk factors [3]. In Cameroon, this knowledge level is also suboptimal, such that 36% of adults are unaware of CV risk factors [21]. Nevertheless, in South Africa, most adults are aware that cigarette smoking and excessive alcohol consumption are risk factors for CVD [24]. The level of education and place of residence have a significant influence on health literacy. It has been reported that higher education levels correlate with a better knowledge of CVD, less number of risk factors and changes in health related behaviour [22, 25].

Gaps in evidence on CVD and risk factors form a barrier to effective prevention of cardiovascular conditions. Thus, evidence on patients’ knowledge of CV risk factors is paramount in primary and secondary prevention of CVD [26]. However, research to reduce the existing evidence gap and the increasing burden of CV risk behaviours in developing countries is scarce. Few studies conducted so far in Ethiopia focussed at describing the high burden of CVD, none of the studies explored CV patients’ knowledge of CV risk factors. Evidence on patients’ knowledge of CV risk factors has vital importance for evidence based health policy and help to design customised interventions. Therefore, the purpose of this study was to assess knowledge of cardiovascular risk factors and associated factors among patients with CVD.

## Methods

### Design, settings and sampling

A cross-sectional survey was conducted in two main referral hospitals in East- Ethiopia, Hiwot Fana Specialised University Hospital and Dilchora Referral Hospital. This study was conducted in chronic follow up units of the two hospitals. The chronic follow up unit provides regular outpatient care for patients with chronic conditions such as hypertension, heart failure, myocardial infarction and diabetes mellitus. The clinic specifically focusses on providing follow up services which include treatment of CVD and counselling of patients to achieve healthy lifestyle behaviours. During the study period (June to September 2018), a total of 820 patients with CVD attended the follow up care in the two participating hospitals.

Patients with a confirmed diagnosis of hypertension, heart failure, or myocardial infarction, in the age range between 18-64yrs were eligible for participation in the study. Patients with congenital heart disorders, rheumatic heart disease, infectious heart disease and inflammatory heart disease were excluded. Mentally ill patients and those with a disability (hearing and talking impairment) which would hinder their ability to participate in the study were also excluded.

The sample size was determined using single population proportion formula with the following assumptions: 95% confidence level, 1.96 (Zα/2), 50% proportion, 5% degree of precision (d), and N (820) total CVD patients attending chronic follow up units of the two hospitals. Based on this assumption and using finite correction, the sample size was 261, and predicting a 10% nonresponse rate, the final sample size was 287. The total 287 calculated sample was allocated for the two hospitals proportional to their total number of patients attending each chronic follow up unit. A convenience sampling was used to select study participants.

Participants were given overview of the study by nurse or physician who were working in follow up unit, then, they were referred to poster information which was posted outside the follow up unit. The poster information contained title of the study, researchers name, eligibility criteria and contact address (mobile phone and email) of data collector. Voluntary participants contacted data collector through phone address or the data collector approached the patients and provided additional information using participant information sheet upon their exit from follow up unit. Recruitment of the patients took place from June to September 2018.

### Ethical considerations

Ethical approval was obtained from the Human Research Ethics Review Committee, University of Adelaide, Australia, and the Institutional Health Research Ethics Review Committee, Haramaya University, Ethiopia before commencing the study. Informed and written consent was obtained from each participant prior to participation in the study.

### Data collection and tools

Data were collected using three validated tools, the World Health Organisation (WHO) STEPs instrument, International physical activity questionnaire and the Heart Disease Fact Questions. The WHO STEPs instrument follows a stepwise approach to chronic disease risk factor surveillance in individuals aged 18–64 years [27]. Ethiopian Public Health Institute adapted the WHO STEPs instrument to Ethiopian context by including khat chewing and the use of local alcohol and cigarette products in the risk behaviour assessment. Locally adapted version of WHO STEPs instrument was translated and used to assess sociodemographic variables and CV risk behaviours including cigarette smoking, alcohol consumption, khat chewing and fruit and vegetable consumption. The international physical activity questionnaire was used to assess physical activity [28].

The primary outcome of the study was knowledge of cardiovascular risk factors among patients with cardiovascular disease. The ‘Heart Disease Fact Questionnaire’ (HDFQ) was used to assess the patient’s knowledge of CV risk factors. The HDFQ showed good content and face validity, and demonstrated adequate internal consistency, with Kuder–Richardson-20 formula of 0.77 [20]. The English version of both the international physical activity questionnaire and the HDFQ were translated into local languages and were back translated into English by language experts to check reliability of the translations. Two nurses who have bachelor qualifications conducted data collection through face to face interviews with patients.

### Measures

Current smoking, khat chewing and alcohol drinking were defined as use within the last 30 days. Inadequate consumption of fruit and vegetables was defined as consumption of less than five servings (equivalent to 400g) of fruit and vegetables per day [27]. Physical activity (PA) level was measured by computing Metabolic Equivalent (MET)-minutes per week for vigorous intensity PA, moderate intensity PA and walking. Vigorous intensity PA was defined as requiring a large amount of effort (>6 METs) and causes rapid breathing and a substantial increase in heart rate. Moderate intensity PA was defined as requiring a moderate amount of effort (3–6 METs) and noticeably acceleration in heart rate. Low level PA was defined as attaining less than 600 MET-minutes per week [29].

Actual cumulative risk behaviour was obtained from the five lifestyle risk behaviours assessed among the patients, (smoking, alcohol drinking, khat chewing, inadequate consumtion of fruit and vegetables and physical inacativity), with a maximum score of 5 (all risk behaviours present) and a minimum score of zero (none of the risk behaviours present).

The patient’s knowledge of CV disease risk was measured using the HDFQ [20] on a two point scale with “0” = wrong answer and “1” = correct answer. Then, it was scored by adding the correct scores of all the items for each participant. A higher score was used to indicate a better knowledge of CV risk factors. The score out of 100 was categorised as good/optimal knowledge (score ≥70%), fair knowledge (score between 50% and 69%) and poor level of knowledge (score <50%). A score < 70% was categorised as suboptimal knowledge [3].

### Statistical analysis

The data was entered on Epidata version 3.0 and were checked for completeness and consistency. Statistical analysis was performed by using IBM SPSS statistics version 25. The univariate analysis was reported as proportion, percentage, and frequency, and continuous data were reported as mean and standard deviation. A normality test was done for continuous variables age and knowledge of CV risk factors. A linear regression model was used to assess association between knowledge of CV risk factors and independent variables. First, associations between knowledge and predictors were analysed by means of bivariate linear regression to identify factors associated with the dependent variable. Then, those variables with a *P*-value < 0.2 on bivariate linear regression were included in a multivariable linear regression model to test for significant associations. The magnitude of the association between different independent variables in relation to the dependent variable was measured using estimates and 95% confidence intervals, and *P*-values < 0.05 were considered to be statistically significant.

## Results

### Characteristics of the participants

A total of 287 patients diagnosed with CVD who attended the chronic follow up care were enrolled in the study; 115 patients from Hiwot Fana Specialised University Hospital and 172 patients from Dilchora Referral Hospital. Mean age was 47 years (±11 SD) and 56.4% of patients were of the female gender. The majority (70.7%) of the patients were diagnosed with hypertension. More than half of the patients had a low level of education. The sociodemographic characteristics of the participants are depicted in Table 1.

**Table 1:** Sociodemographic characteristics of patients attending chronic follow up care in eastern Ethiopia, 2018.

**Variable (N = 287)**	
Mean age ± SD	47 years ±11
**Variable (N = 287)**	**N (%)**
Sex	
Female	162 (56.4)
Clinical characteristics	
Hypertension	203 (70.7)
Heart failure	77 (26.8)
Hypertension and heart failure comorbid	6 (2.1)
Myocardial infarction	1 (0.4)
Residence	
Urban	259 (90.2)
Level of education	
No formal education	81 (28.2)
Less than primary school	72 (25.1)
Primary school completed	52 (18.1)
Secondary school completed	50 (17.4)
College or university completed or postgraduate	32 (11.1)
Ethnicity	
Oromo	90 (31.4)
Amhara	160 (55.7)
Somali	10 (3.5)
Tigray	10 (3.5)
Harari	3 (1)
Gurage/Silte	14 (4.9)
Marital status	
Currently married	228 (79.4)
No longer married	38 (13.2)
Never married	21 (7.3)

### Knowledge of cardiovascular risk factors

**Fig 1:**
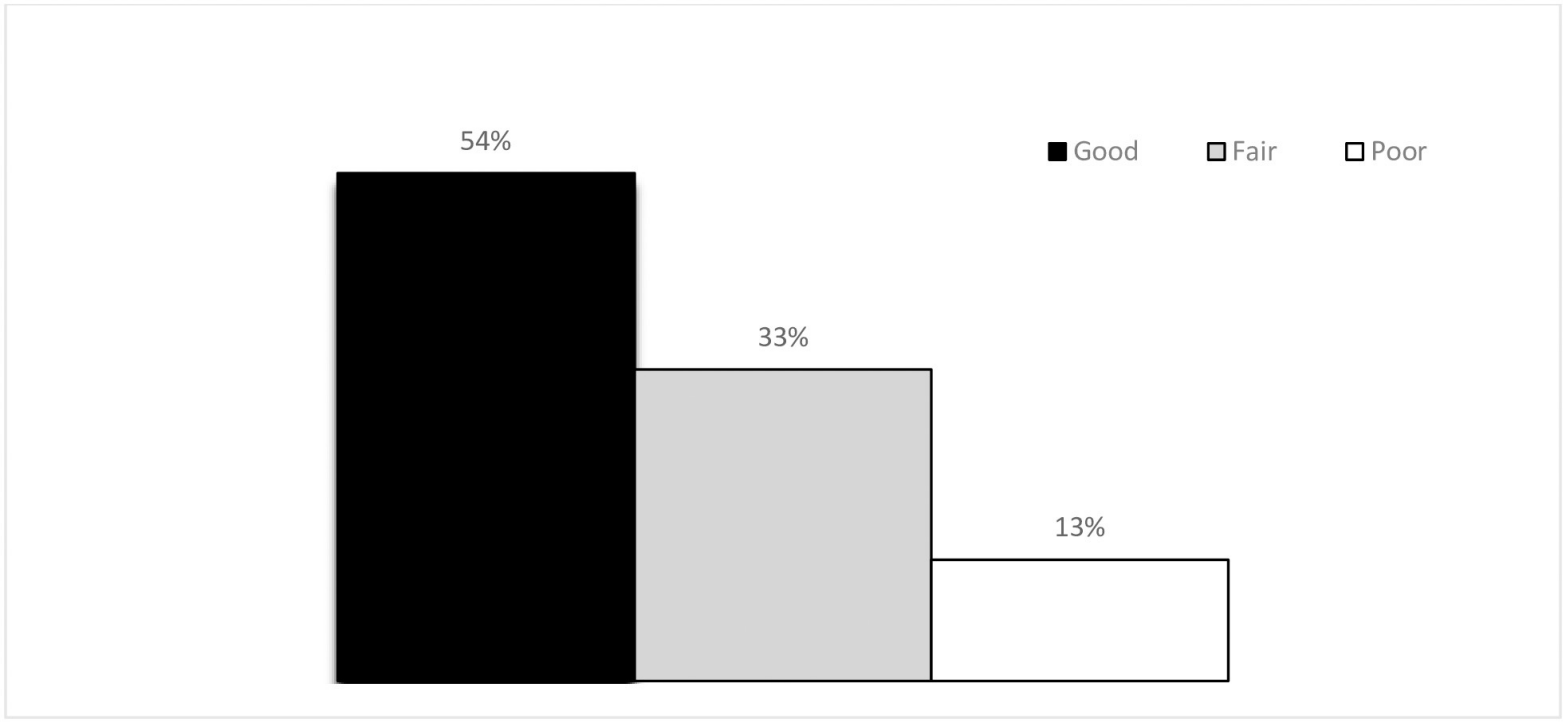
Knowledge of risk factors among CVD patients attending chronic follow up care.

The mean percentage HDFQ score was 70.5% (±15.3). Overall, 155 patients (54%) had optimal knowledge of risk factors (scored ≥70%), whereas, the remaining 132 patients (46%) had sub-optimal knowledge (Fig 1). The majority of patients demonstrated significant knowledge about facts that age, 228 (79.4%), smoking 280 (97.6%), being overweight 262 (91.3%) and high blood pressure 235 (81.9%) are risk factors for cardiovascular disease. At the same time patients had deficient knowledge about the fact that family history of heart disease 249 (86.8%) and diabetes 184 (64.1%) are also risk factors. Almost one fifth 55 (19.2%) did not understand that keeping blood pressure under control reduces the risk of developing cardiovascular disease, 52 (18.1%) were unable to identify eating fatty food affects blood cholesterol level, and 115 (40.1%) assume only exercising at a gym or in an exercise class lower a chance of developing cardiovascular disease. Table 2 shows the percentage of patients who answered the heart disease fact questions correctly.

**Table 2:** Responses to the HDFQ among patients attending chronic follow up care in eastern Ethiopia, 2018.

Questions (n = 287)	Correct response	Frequency N (%)
A person always knows when they have heart disease	False	128 (44.6)
If you have a family history of heart disease, you are at risk for developing heart disease	True	38 (13.2)
The older a person is, the greater their risk of having heart disease	True	228 (79.4)
Smoking is a risk factor for heart disease	True	280 (97.6)
A person who stops smoking will lower their risk of developing heart disease	True	256 (89.2)
High blood pressure is a risk factor for heart disease	True	235 (81.9)
Keeping blood pressure under control will reduce a person’s risk for developing heart disease	True	232 (80.8)
High cholesterol is a risk factor for developing heart disease	True	247 (86.1)
Eating fatty foods does not affect blood cholesterol levels	False	235 (81.9)
If your ‘good’ cholesterol (HDL) is high you are at risk for heart disease	False	73 (25.4)
If your ‘bad’ cholesterol (LDL) is high you are at risk for heart disease	True	220 (76.7)
Being overweight increases a person’s risk for heart disease	True	262 (91.3)
Regular physical activity will lower a person’s chance of getting heart disease	True	264 (92)
Only exercising at a gym or in an exercise class will lower a person’s chance of developing heart disease	False	172 (59.9)
Walking and gardening are considered exercise that will help lower a person’s chance of developing heart disease	True	265 (92.3)
Diabetes is a risk factor for developing heart disease	True	103 (35.9)

### Actual cumulative risk behaviour and knowledge of cardiovascular disease risk factors association

Through our previous study [30], we have assessed five CV risk behaviours, i.e. smoking, alcohol drinking, khat chewing, fruit and vegetable intake and physical activity. None of the patients met the WHO recommendation for fruit and vegetable consumption (more than five serving daily), 148 (51.6%) were physically inactive (attained less than 600 MET-min per week), 57 (19.9%) were current khat chewers, 54 (18.8%) were current alcohol drinkers and 3 (1%) were current smokers. Almost one-third 86 (30%) them had one risk behaviour, more than half 149 (51.9%) had two risk behaviours, and 43 (18.1%) had three or more risk behaviours. Out of the total recruited patients, 201 (70%) had multiple risk behaviours (two or more behaviours).

Regarding bivariate linear regression analysis age, sex, residence, ethnicity, marital status, education level and number of actual risk behaviours got p<0.2 (Table 3). These variables were taken in to multivariable linear regression model to identify independent predictors of CV risk factors knowledge.

**Table 3:** Bivariate linear regression analysis of associated factors of knowledge of CV risk factors in eastern Ethiopia, 2018.

Variables	β	95% Wald Confidence Interval	P-value
Age	0.13	(-0.01, 0.29)	0.080
Sex			
Male	3.51	(-0.26, 7.06)	0.052
Female ^R^			
Residence			
Urban	16.30	(10.64, 21.95)	<0.001
Rural ^R^			
Ethnicity			
Oromo	1.42	(-7.00, 9.85)	0.740
Amhara	4.17	(-2.02, 14.33)	0.140
Somali	2.67	(-9.46, 14.82)	0.666
Tigray	14.55	(2.40, 26.70)	0.019
Harari	9.52	(-22.23, 15.09)	0.708
Gurage/Silte ^R^			
Marital status			
Never married	-11.97	(-18.65, -5.28)	<0.001
No longer married	-3.04	(-8.18, 2.09)	0.246
Currently married ^R^			
Education level			
No formal education	-19.29	(-25.01, -13.57)	<0.001
Less than primary	-11.93	(-17.75, -6.11)	<0.001
Primary school completed	-9.16	(-15.32, -3.00)	0.004
Secondary school completed	-5.89	(-12.09, 0.31)	0.063
College or university completed or postgraduate ^R^			
Number of actual risk behaviours			
One	1.08	(-9.38, 11.54)	0.839
Two	0.87	(-9.37, 11.12)	0.067
Three	-2.69	(-13.64, 8.25)	0.629
Four ^R^			

R-Reference, β- Beta coefficient, variables with P-value <0.2 included in multivariate linear regression

In the multivariable linear regression analysis, knowledge of CV risk factors was significantly associated with place of residence, level of education and marital status. There was a statistically significant association between knowledge of CV risk factors and residence (P < 0.001). Urban residents had 12.84 units higher mean knowledge score than rural residents (β = 12.84, 95% CI 6.91 to 18.77; P < 0.001). In addition, level of education is associated with knowledge of CV risk factors (P < 0.001), those who had no formal education had -18.80 units lower mean knowledge score compared to those who completed college or university (β = -18.80, 95% CI -24.76 to -12.85; P < 0.001). Those who attained less than primary school education had -12.02 units less knowledge score compared to those who completed college or university (β = -12.02, 95% CI -17.63 to -6.40; P < 0.001). There was also a statistically significant association between knowledge and marital status (P < 0.001). Those who were never married had -14.01 units lower mean knowledge score than those who were currently married (β = -14.01, 95% CI -20.71 to -7.29; P < 0.001). There was no statistically significant association between knowledge of CV risk factors and actual cumulative risk behaviour (P = 0.076) or age (P = 0.718) or sex (P = 0.259) or ethnicity (P = 0.196) (Table 4).

**Table 4:** Multivariable analysis of potential associations of CV risk factors knowledge with health behaviour and sociodemographic variables in eastern Ethiopia, 2018.

Variables	β	95% Wald Confidence Interval	P value
Age	-0.03	(-0.20, 0.14)	0.718
Sex			
Male	2.08	(-1.53, 5.70)	0.259
Female ^R^			
Residence			
Urban	12.84	(6.91, 18.77)	**<0.001**
Rural ^R^			
Ethnicity			
Oromo	6.11	(-1.39, 13.62)	0.110
Amhara	4.78	(-2.52, 12.08)	0.200
Somali	5.78	(-4.96, 16.51)	0.292
Tigray	14.26	(3.26, 25.27)	0.101
Harari	2.96	(-13.54, 19.57)	0.727
Gurage/Silte ^R^			
Marital status			
Never married	-14.01	(-20.71, -7.29)	**<0.001**
No longer married	-0.82	(-5.52, 3.86)	0.729
Currently married ^R^			
Education level			
No formal education	-18.80	(-24.76, -12.85)	**<0.001**
Less than primary	-12.02	(-17.63, -6.40)	**<0.001**
Primary school completed	-10.63	(-16.35, -4.91)	**<0.001**
Secondary school completed	-7.56	(-13.33, -1.80)	0.110
College or university			
Completed or postgraduate ^R^			
Number of actual risk behaviours			
One	4.98	(-4.00, 13.97)	0.277
Two	7.38	(-1.48, 16.25)	0.103
Three	2.25	(-7.16, 11.67)	0.639
Four ^R^			

R = Reference, β- Beta coefficient, P-value <0.05 is statistically significant

## Discussion

This study examined the level of knowledge of cardiovascular risk factors and associated factors among known CV patients who were attending chronic follow up care at two public referral hospitals in eastern Ethiopia. The study demonstrates that almost half of CVD patients have suboptimal knowledge regarding CV risk factors which may impede secondary CV prevention if effective interventions are not implemented. Thus, the findings of this study warrant the need of improved preventive interventions to achieve optimal knowledge in the general population.

Knowledge of CV risk factors among CVD patients was unsatisfactory, and about half of the patients have suboptimal knowledge, which is in line with existing findings reported from India and United Arab Emirates [16, 31]. However, the mean CV risk factors knowledge score in the current study (70.5%) is higher compared to finding from Nigeria (48.6%), and this could be due to difference in population characteristics [3]. Consistent with the finding of this study, a systematic review showed low level knowledge and awareness of CVD and associated risk factors among populations in Sub-Saharan Africa [22]. The possible reasons for the suboptimal knowledge may be attributed to a lower level of educational attainment of the patients, poor patient counselling during follow up care appointment and absence of intensive lifestyle counselling programs. Moreover, low health literacy may be due to lack of effective patient counselling methodologies that fits the cultural and sociodemographic context and poor health information seeking behaviour of patients. Implementation of innovative health education strategies may help to improve health literacy for CV patients and for the general population as well.

Residence, education level and marital status were associated with knowledge of cardiovascular risk factors, which mirrors that social, cultural and economic factors are major determinants of awareness and health behaviour change [32]. In line with the finding of the current study, numerous studies [25, 33, 34] have revealed higher education is associated with better health literacy. A review conducted in Sub-Saharan Africa reported that place of residence is an important determinant of knowledge of cardiovascular risk factors, i.e. urban residence is associated with improved knowledge of CV risk factors [22]. In Ethiopia, rural residents attain lower educational level and have poor access to health information as compared to urban residents who relatively have better health literacy. Thus, low knowledge of CV risk factors in rural residents could be due to their lower education attainment. Moreover, the current study shows that those who were never married have lower levels of knowledge regarding CV risk factors compared to those who were married. Consistent with this, Manfredini et al. reported that being married is associated with, lower risk factors, better knowledge and better CV health status [35].

Studies from Nigeria, Germany and Luxembourg reported that a higher level of education is associated with healthy lifestyle and appropriate self-care behaviours [25, 36, 37]. In addition, evidence from a review revealed that a lower educational level is associated with lower knowledge of CV risk factors, and this also concurs with the finding of the current study [22]. Findings from Pakistan also support those of this study where lack of formal education is associated with lower knowledge of cardiovascular disease risk factors [38]. However, about one-third of the patients in the current study had no formal education, thus, improving literacy in developing countries is vital in tackling the emerging burden of chronic diseases, in particular, CVD and its associated lifestyle behaviours, as demonstrated previously [22].

The prevalence of alcohol drinking, inadequate fruit and vegetable consumption and physical inactivity in the current study is comparable to findings from Addis Ababa [14], Kenya [39] and Nigeria [40]. However, the rate of smoking in this study is lower compared to findings from Addis Ababa [14], Ghana [41], Kenya [42] and Uganda [43], and this could be due to differences in sociocultural characteristics of participants.

According to the Health Belief Model, knowledge of health behaviour is an important determinant of adherence to healthy lifestyle behaviours. Though, knowledge alone is not sufficient, and patients’ perceptions and attitudes of health behaviours are also important predictors of health lifestyle behaviours. The current study demonstrated that occurrence of actual cumulative risk behaviours is not associated with knowledge of CV risk factors. Thus, as patients’ perceptions and attitudes of CV risk factors are important determinants of behaviour change, these need to be explored in further research. Consolidating this, Tran et al (2017) states a high level of knowledge of CV risk factors is not sufficient to reduce cardiovascular risk, however, improving the perception of adults regarding CV risk factors plays an important role in reducing long term cardiovascular risk [23]. Nevertheless, the finding of Alzaman et al. which states awareness of modifiable CV risk factors is positively associated with health behaviour for adult patients [44] is inconsistent with the finding of the current study. A potential reason may be due to differences in education profile.

Even though the overall actual risk behaviour is not associated with occurrence of actual cumulative risk behaviour, the vast majority of patients had good knowledge and practice healthy behaviour regarding smoking. Available evidence reports that most adults are aware of the fact that cigarette smoking is a risk factor for CV disease [22, 24, 45]. Inadequate consumption of fruit and vegetables was highly and equally (100%) prevalent among those who have good or fair or poor level knowledge of CV risk factors. In addition, the majority of patients knew physical activity lowers the chance of developing heart disease, however, more than half of them failed to achieve this. This shows the existence of other factors that determine patients’ health behaviours, including individual perceptions and beliefs regarding the disease and the risk factors. This issue needs to be explored more through further research.

Findings show intensive lifestyle counselling improves awareness and adherence to healthy lifestyle behaviours [25, 26, 46]. In the current study, about half of CVD patients who had received follow up care with a focus on the management of CV risk factors had sub-optimal knowledge of these and they were indulged in multiple unhealthy behaviours. This is consistent with findings from America which reported African Americans have cluster of CV risk behaviours [47]. In addition, about one fifth do not know high blood pressure is a risk factor for heart disease, and this indicates a need for implementing targeted education strategies. Overall, the finding of this study show existing follow-up service is not optimal, and the probable reasons for this may be poor patient counselling service and limitation of resources. This signifies there is a need to improve the follow up service to promote healthy lifestyle behaviours for the patients. Implementing intensive lifestyle support programs based on developed guidelines and delivered by trained health professionals may also help to improve patients’ knowledge and health behaviours [48, 49]. Absence of CVD prevention policies and strategies at population level could also have contributed to this problem in Ethiopia. Various CVD prevention guidelines have been developed and are in use to promote effective prevention of CVD in developed countries. The European Society of Cardiology guidelines focus on the importance of patient involvement and patient education which may potentially improve knowledge levels and motivation in patients [50]. The American College of Cardiology (ACC) and the American Heart Association (AHA) guidelines recommend promotion of lifetime risk estimation and which may represent an additional step forward in supporting lifestyle behaviour change counselling programs [51]. Other than a recently developed *National Strategic Action Plan (NSAP) for prevention & control of non-communicable diseases* [52], there are no specific guidelines for prevention of CVD in use in Ethiopia. Therefore, there is a need for the development and implementation of context specific guidelines and innovations to improve knowledge levels and patient motivation towards healthy lifestyle behaviour, particularly for poorly educated and rural residents.

Adoption of healthy lifestyle behaviours promote better health related quality of life [53], however, the patients in the current study had unhealthy behaviours that may predispose them for further complications and affect their health related quality of life, and this may contribute to the increased CVD related mortality in Ethiopia. Despite the rise in the burden of CV risk factors and lack of awareness among adult population, there is no prevention strategy implemented to reduce the burden of CVD in Ethiopia. The findings of this study have practical implications for health care workers and should inform policy makers that change is required to improve patients’ understanding of cardiovascular disease risk factors and reduce the burden of CV risk behaviours.

Given that the actual risk behaviour is not associated with the required knowledge of risk factors in this population, warrants the design and implementation of innovative interventions, in which patients are educated and empowered to self-manage their risk factors. As an example, structured and systematic nurse-led lifestyle counselling effectively reduce cardiovascular risk behaviour, improve patients’ knowledge of CV risk factors and promote healthy lifestyle behaviours [46]. Moreover, health care providers should identify patients with limited understanding of risk factors and actual risk behaviours and provide tailored interventions. Indeed, it is essential to explore how patients perceive their own risk of CV disease and the risk factors, since these are key determinants of health behaviour change according to the Health Belief Model. Therefore, the findings of this study warrant attention and are a call for action from policy makers. As such the presented data can be used as baseline data for the development of intervention programs, specifically focussed at Ethiopia that aim to improve patients’ awareness of CV disease risk factors and reduce the burden of CV risk behaviours. Indeed, it is important to design and implement monitoring and evaluation systems to improve the follow up service.

## Limitations

This study may be subject to bias. Firstly, the study is subject to the limitations of patient recall and social desirability bias, and the self-reported measurement of risk behaviours may have underestimated the CV risk behaviours. However, this underlines that the real-world problem may be even worse in developing countries, and that a call for action is required. Secondly, the use of cross-sectional study design does not establish causal relationships.

## Conclusion

The burden of CV risk behaviours is increasing whilst the patients’ understanding of associated risk factors is limited. Almost half of CVD patients have suboptimal knowledge regarding CV disease risk factors, and they have multiple unhealthy behaviours though they attend chronic follow up care clinics. Lower education, rural residence and single marital status were associated with lower knowledge of cardiovascular risk factors. Therefore, this study is important to demonstrate the need for implementing an effective prevention program. In line with intensive patient counselling and education to improve awareness regarding CV risk factors, implementation of multidisciplinary, innovative interventions and systematic nurse-led lifestyle counselling is indeed important to effectively assist CV patients in adopting positive lifestyle behaviours. Moreover, implementation of CVD prevention programs should be considered for the disease prevention policy agenda in Ethiopia.

## Supporting information

S1 Checklist STROBE statement—Checklist of items that should be included in reports of *cross-sectional studies*. (DOCX)
